# Characterization of the Newly Developed Soybean Cultivar DT2008 in Relation to the Model Variety W82 Reveals a New Genetic Resource for Comparative and Functional Genomics for Improved Drought Tolerance

**DOI:** 10.1155/2013/759657

**Published:** 2012-12-27

**Authors:** Chien Van Ha, Dung Tien Le, Rie Nishiyama, Yasuko Watanabe, Uyen Thi Tran, Nguyen Van Dong, Lam-Son Phan Tran

**Affiliations:** ^1^Signaling Pathway Research Unit, Plant Science Center, RIKEN Yokohama Institute, 1-7-22 Suehiro-cho, Tsurumi, Yokohama 230-0045, Japan; ^2^National Key Laboratory of Plant Cell Biotechnology, Agricultural Genetics Institute, Vietnamese Academy of Agricultural Science, Pham Van Dong Street, Hanoi, Vietnam; ^3^Post-Graduate Program, Vietnamese Academy of Agricultural Science, Thanhtri, Hanoi, Vietnam

## Abstract

Soybean (*Glycine max*) productivity is adversely affected by drought stress worldwide, including Vietnam. In the last few years, we have made a great effort in the development of drought-tolerant soybean cultivars by breeding and/or radiation-induced mutagenesis. One of the newly developed cultivars, the DT2008, showed enhanced drought tolerance and stable yield in the field conditions. The purpose of this study was to compare the drought-tolerant phenotype of DT2008 and Williams 82 (W82) by assessing their water loss and growth rate under dehydration and/or drought stress conditions as a means to provide genetic resources for further comparative and functional genomics. We found that DT2008 had reduced water loss under both dehydration and drought stresses in comparison with W82. The examination of root and shoot growths of DT2008 and W82 under both normal and drought conditions indicated that DT2008 maintains a better shoot and root growth rates than W82 under both two growth conditions. These results together suggest that DT2008 has better drought tolerance degree than W82. Our results open the way for further comparison of DT2008 and W82 at molecular levels by advanced omic approaches to identify mutation(s) involved in the enhancement of drought tolerance of DT2008, contributing to our understanding of drought tolerance mechanisms in soybean. Mutation(s) identified are potential candidates for genetic engineering of elite soybean varieties to improve drought tolerance and biomass.

## 1. Introduction

Soybean (*Glycine max*) is one the world's leading economic oilseed crops, providing the largest source of vegetable oil, proteins, macronutrients, and minerals for human consumption and animal feed. The consumption of soybean as food products is increasing worldwide because of its beneficial effects including lowering of cholesterol and prevention of cancer, diabetes, and obesity [1–4]. In addition, soybean is also an attractive crop for the production of biodiesel which produces more usable energy and less greenhouse gases than corn-based ethanol [5]. 

Vietnam with its 161.200 ha of soybean cultivated land produces approximately 235.450 metric tonnes (~1.41 metric tonnes/ha in average as recorded for the year of 2009) which is sufficient for only 8–10% of soybean consumption in the country [6]. The low productivity of soybean in Vietnam is mainly attributed to drought stress [6] which also affects soybean production worldwide [1, 7]. Therefore, in the last decade the Vietnamese soybean research community has spent much effort on the development of drought-tolerant soybean cultivars. Several elite soybean cultivars were obtained by using conventional breeding coupled with radiation-induced mutagenesis. One of the varieties, the DT2008, exhibited enhanced tolerance to various biotic and abiotic stresses, including drought, enabling the farmers to grow the DT2008 cultivar in three crops/year with high and stable yield across the country (~2–4 metric tonnes/year depending on the regions) [6].

Plants, including soybean, activate various mechanisms to cope with drought stress [8–10]. In the last 20 years, many genes, including both regulatory and functional genes, have been identified in important crops, such as rice (*Oryza sativa*) and soybean, to be involved in defence mechanisms that function to increase drought tolerance [7, 11–17]. One of the preferred approaches widely used to identify stress-responsive genes was large-scale expression profiling using microarray analysis or high-throughput qRT-PCR [18–23]. The genomic sequence of the model soybean cultivar Williams 82 (W82) has been completed two years ago [24], enabling us to carry out large-scale expression profiling of soybean gene families [15, 20] or design gene chips for genome-wide identification of drought-responsive genes [21, 25]. With the advance in proteomic and metabolomic technologies, an increasing number of literature has described the applications of these techniques in study of mechanisms and signalings related to drought responses in soybean [26, 27]. 

In this work, we compared the drought-tolerant phenotypes of DT2008 and W82 by examining the dehydration-induced water loss and membrane stability of the shoot parts of the young seedlings. The results suggest that the enhanced drought tolerance of DT2008 is, at least in part, associated with shoot traits. Further comparisons of shoot and root growth under normal and drought stress conditions indicated that DT2008 grew better than W82 under both conditions. Data on root growth suggest that the better drought tolerance of DT2008 is coincided with better root growth and development in comparison with W82. The differential drought-responsive phenotypes of DT2008 and W82 will enable us to identify mutation(s) which caused drought-tolerance of DT2008 using systems biology-based approaches, such as transcriptomics, proteomics, or metabolomics. The mutations and/or mutated gene(s) identified can be used to improve drought tolerance of soybean cultivars, which have high productivity but are sensitive to drought stress, by genetic engineering.

## 2. Materials and Methods

### 2.1. Measurement of Relative Water Content under Dehydration

DT2008 and W82 seeds were separately germinated in 6-litre pots (10 seeds/pot) containing vermiculite and grown under well-water conditions in greenhouse (continuous 30°C temperature, photoperiod of 12 h/12 h, 80 *μ*mol m^−2^ s^−1^ photon flux density, and 60% relative humidity). Ten-day-old DT2008 and W82 plants were carefully removed from soil, and roots were gently washed to remove soil. The plants were subsequently transferred onto a filter paper and allowed to dry for 5 h under the following conditions: 60% relative humidity, 25°C temperature and 10 *μ*mol m^−2^ s^−1^ photon flux light intensity. Dehydrated shoot samples (without cotyledon leaves and root) were individually weighed to determine sample weight (W) after 5 h of dehydration (*n* = 20). Individual dehydrated samples were then placed into 50 mL tubes and hydrated overnight in 40 mL of deionized water to full turgidity under normal room light and temperature. The samples were then removed from water, residual leaf moisture was gently removed with filter paper, and samples were immediately weighed to obtain a fully turgid weight (TW). Subsequently, the plants were dried in an oven at 65°C for 48 h, and dry weight was measured (DW). RWC was calculated as RWC (%) = [(W − DW)/(TW − DW)] × 100 [28]. 

### 2.2. Measurement of Relative Water Content under Drought Stress

Two DT2008 and two W82 seeds were germinated in each 6-litre pot containing vermiculite and grown under well-water conditions in greenhouse (continuous 30°C temperature, photoperiod of 12 h/12 h, 80 *μ*mol m^−2^ s^−1^ photon flux density and 60% relative humidity). For drought treatment, water was withheld from 5-day-old plants for 15 or 20 days. Volumetric soil moisture contents were monitored at 5-day intervals during drought stress treatment using a Hydrosense soil moisture probe (Campbell Scientific Inc.) (*n* = 5). For control, plants were grown in parallel under well-watered conditions. Detached aerial parts of stressed plants (*n* = 10) were individually weighed to determine sample weight (W) after 15 or 20 days of water withholding. After the initial determination of the sample fresh weight, all the samples were fully dipped in deionized water overnight under normal room light and temperature for rehydration to full turgidity. The samples were then removed from water, residual leaf moisture was gently removed with filter paper, and samples were immediately weighed to obtain a fully turgid weight (TW). Subsequently, the plants were dried in an oven at 65°C for 48 h, and dry weight was measured (DW). RWC was calculated as RWC (%) = [(W − DW)/(TW − DW)] × 100 [28].

### 2.3. Root and Shoot Growth Assays of 5-D-Old and 10-D-Old Young Seedlings under Well-Watered Conditions

DT2008 and W82 seeds were separately germinated in 6-litre pots (10 seeds/pot) containing vermiculite and grown for 5 or 10 days under well-watered conditions in greenhouse as described in Section 2.1. The 5- and 10-day-old seedlings were carefully removed from soil, and roots were gently washed to remove soil. The fresh weight (FW) of shoot part of each seedling was separately measured, and the length of the shoot and primary root of each plant was also determined. The number of the secondary roots of the 5-day-old plants was also counted. Subsequently, all the shoot and root samples were individually dried in an oven at 65°C for 48 h, and the dry weight (DW) of each root or shoot sample was measured (*n* = 20). 

### 2.4. Root and Shoot Growth Assays of 20-D-Old and 25-D-Old Soybean Plants under Normal and Drought Conditions

DT2008 and W82 plants were grown, and drought stress treatment was performed as described in Section 2.2. The number of the trifoliate leaves of well-watered and stressed plants was counted at 5-day intervals during growth. For further comparison of root and shoot growths under drought stress, plants were carefully removed from soil, and roots were gently washed to remove soil after the drought treatment. The FW of shoot part of the well-watered and stressed plants was measured, and the length of the shoot of each plant was also determined (*n* = 10). Subsequently, the shoot and root samples were individually dried in an oven at 65°C for 48 h, and dry weight of each root or shoot sample was measured.

### 2.5. Statistical Analysis of the Data

Average values were used to plot figures, and error bars on each figure represent the standard errors. When appropriate, a Student's *t*-test (one-tailed, unpaired, and equal variance) was used to determine the statistical significance [20]. 

## 3. Results and Discussion

### 3.1. DT2008 Variety Has Higher RWC than the Model W82 Variety under Dehydration and Drought Stress Conditions

DT2008 soybean variety was produced in Vietnam by multiple hybridizations of local varieties and subsequent irradiation with Co^60^. The significantly improved drought tolerance and productivity as well as yield stability of DT2008 have been examined in various regions in Vietnam in comparison with other local elite soybean cultivars which were used in production [6]. DT2008, therefore, is an important biological resource which can be used for the identification of mutations involved in the regulation of drought tolerance. Thus, in this study, we compared the drought-tolerant phenotypes of DT2008 and W82 to examine whether these two cultivars possess differential drought-tolerant phenotype, thereby providing research materials for identification of potential drought-related mutations in DT2008. These mutations may cause differential gene and protein expression or altered metabolomic pathways that can be identified using advanced omic approaches, such as transcriptomics, proteomics, or metabolomics. W82 was chosen because it is a soybean model plant whose genome sequence has been recently completed [24]. Thus, a large amount of genetic data has been available for this species, providing a basic foundation for comparative genomics of DT2008 and W82.

**Figure 1:**
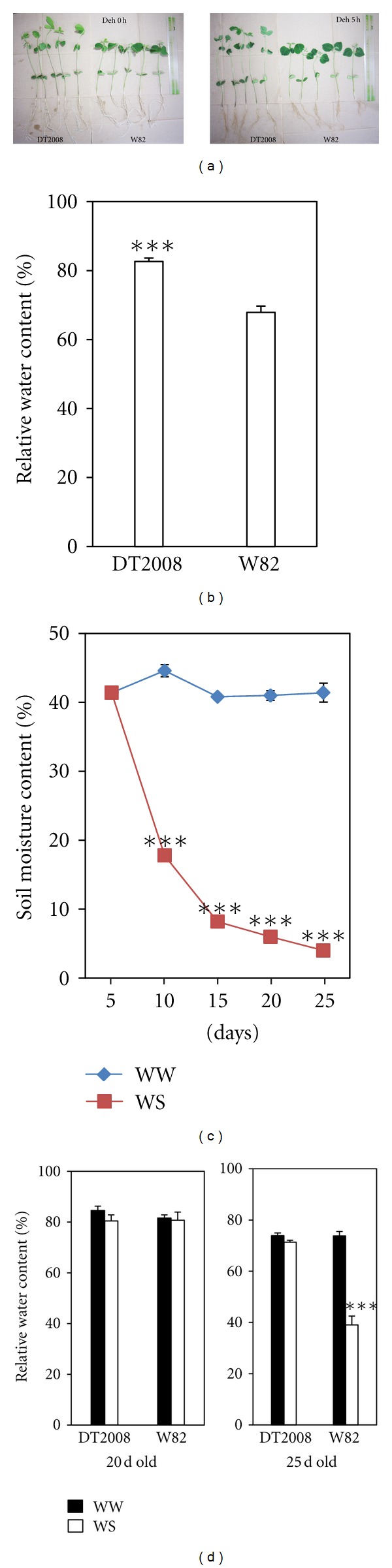
Relative water content (RWC) of DT2008 and W82 plants. (a) Ten-day-old plants were grown and exposed to dehydration stress as described in Section 2. (b) After 5 h of dehydration treatment, RWC was measured. Error bars represent standard errors (*n* = 20 plants/genotype). (c) Volumetric soil moisture contents were monitored during the drought stress treatment (*n* = 5). (d) Five-day-old plants were grown in pots and exposed to drought stress. After 15 or 20 days of water withholding, RWC was measured. Error bars represent standard errors (*n* = 10 plants/genotype). WW, well-watered control; WS, water stress. Asterisks indicate significant differences as determined by a Student's *t*-test (***P < 0.001).

The drought-tolerant phenotypes of DT2008 and W82 were compared by means of comparing their water loss under dehydration or drought stress conditions. When 10-day-old plants were dehydrated as shown in Figure 1(a), the DT2008 seedlings could maintain higher RWC under dehydration in comparison with W82 plants (Figure 1(b)). When both DT2008 and W82 plants were subjected to a soil drying experiment (SMC was reduced to below 10%, Figure 1(c)), DT2008 plants had significantly lower water loss rate than W82 plants as well, specifically after 20 days of water withholding (Figure 1(d)). These results suggest that DT2008 has capacity to be tolerant better to drought stress than W82.

### 3.2. Differential Shoot Growth of DT2008 and W82 under Normal and Drought Conditions

The drought-tolerant phenotypes of DT2008 and W82 were also compared by evaluating their shoot growth under normal and drought stress conditions. For well-watered conditions, the length, FW and DW of the shoots of 5-, 10-, 20- and 25-day-old seedlings grown in soil pots were assessed under well-watered conditions. For drought stress treatment, seedlings were grown in pots for 5 day under well-watered conditions, subsequently subjected to 15 or 20 days of water-withholding, then the same growth parameters were measured. For both well-watered and drought stress conditions, the numbers of the trifoliate leaves of DT2008 and W82 plants were also counted at 5-day intervals for comparison.

**Figure 2:**
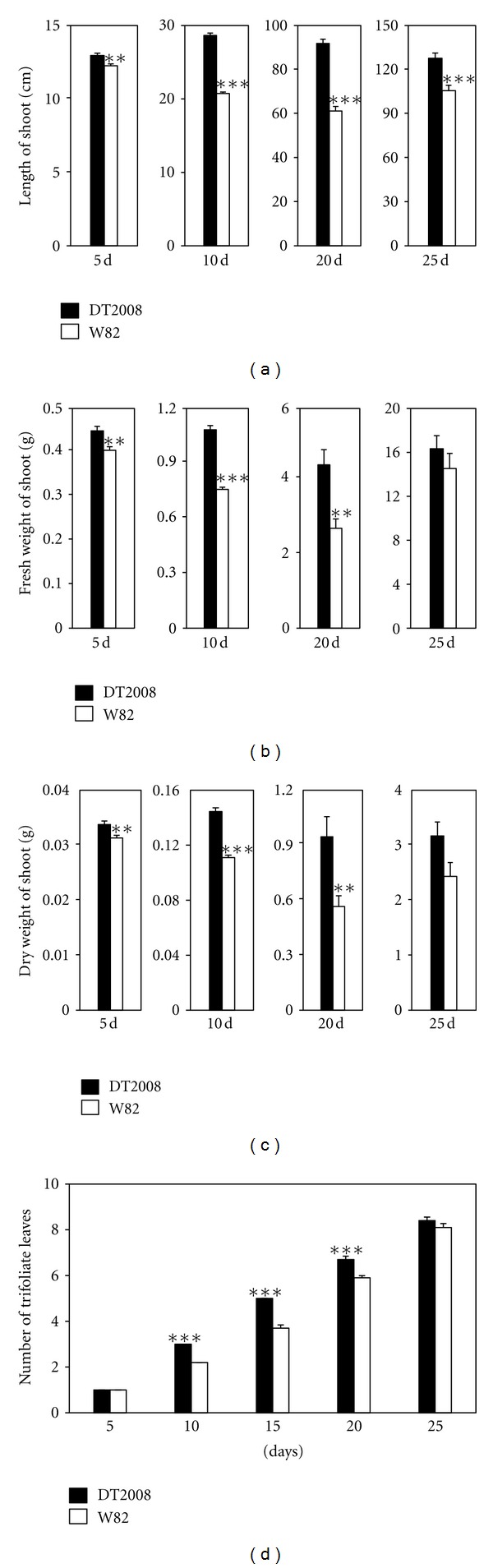
Comparison of shoot growth of DT2008 and W82 plants under well-watered conditions. (a) Comparison of shoot lengths of DT2008 and W82 plants after growth in pots for 5, 10, 20, and 25 days. (b) Comparison of fresh weights of 5-, 10-, 20-, and 25-day-old DT2008 and W82 plants. (c) Comparison of dry weights of 5-, 10-, 20- and 25-day-old DT2008 and W82 plants. For 5- and 10-day-old plants, *n* = 20 plants/genotype. For 20- and 25-day-old plants, *n* = 10 plants/genotype. (d) Comparison of numbers of trifoliate leaves of DT2008 and W82 plants at indicated time points (*n* = 10). Error bars represent standard errors. Asterisks indicate significant differences as determined by a Student's *t*-test (*P < 0.05; **P < 0.01; ***P < 0.001).

Under normal growth conditions we observed that the DT2008 plants exhibited higher shoot growth and more trifoliate leaves than W82 (Figure 2), suggesting that DT2008 possesses better shoot growth rate than W82. Thus, DT2008 is a potential variety for comparative genomics to identify genes or SNPs (single nucleotide polymorphisms) involved in regulation of shoot biomass. The increase in biomass production can be exploited as a mechanism to enhance plant productivity because increased yields have been shown to be associated with improved biomass production [29, 30].

**Figure 3:**
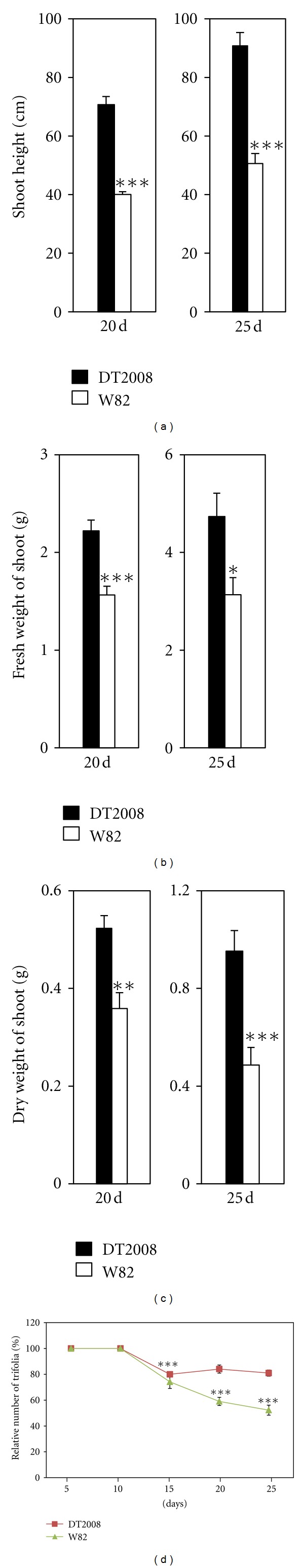
Comparison of shoot growth of DT2008 and W82 plants under drought stress. (a) Five-day-old DT2008 and W82 plants grown in pots were exposed to 15 or 20 days of water withholding, and their shoot lengths were compared. (b) Five-day-old DT2008 and W82 plants grown in pots were exposed to 15 or 20 days of water withholding, and their shoot fresh weights were compared. (c) Five-day-old DT2008 and W82 plants grown in pots were exposed to 15 or 20 days of water withholding, and their shoot dry weights were compared. (d) Numbers of the trifoliate leaves of well-watered and stressed DT2008 and W82 plants were counted at 5-day intervals during growth to determine relative number of trifolia at indicated time points for comparison. Error bars represent standard errors (*n* = 10 plants/genotype). Asterisks indicate significant differences as determined by a Student's *t*-test (*P < 0.05; **P < 0.01; ***P < 0.001).

As for comparison of shoot growth of DT2008 and W82 under drought stress conditions, we found that drought stress more drastically inhibited the growth of W82 than that of DT2008 as shown by higher decreases in the length, FW and DW of shoot as well as the numbers of trifoliate leaves observed for W82 when compared with DT2008 (Figure 3). These data further support that DT2008 is more strongly tolerant to drought stress than W82. Drought stress inhibits shoot growth perhaps by limiting photosynthesis as reported previously [27]. Our result suggests that comparative and functional genomics of DT2008 and W82 will enable us to identify mutations responsible for improved biomass, thereby yield, under adverse conditions.

### 3.3. Differential Root Growth of DT2008 and W82 under Normal and Drought Conditions

Strong lines of evidence suggest that the degree of drought tolerance is positively correlated with root traits [1, 13]. Because the distribution of water within the rhizosphere is critical to maintaining function in different environmental conditions, an enhanced root development is an essential trait for drought tolerance. A long taproot will enable plants to reach lower soil layers where deep water is available, thereby helping plants adapt better to drought stress. An extensive secondary root system will allow plants to forage subsoil surface moisture [1]. Therefore, the root trait is a promising target for breeding or genetic engineering to develop improved drought-tolerant crops, including soybean [1, 28]. 

**Figure 4:**
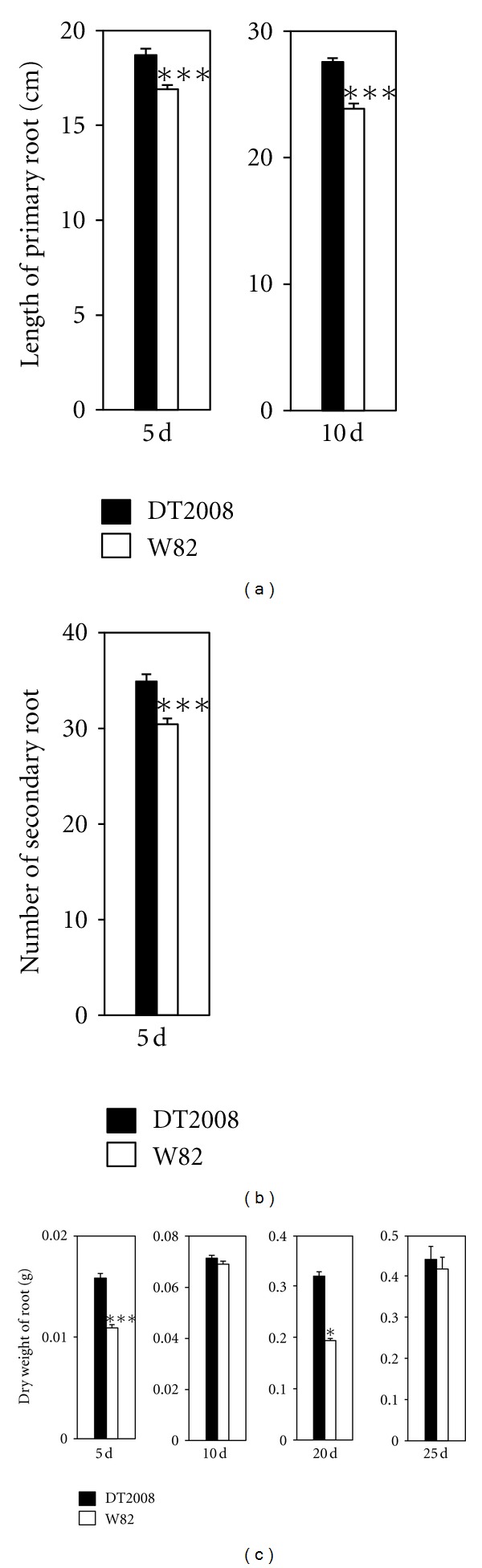
Comparison of root growth and development of DT2008 and W82 plants under well-watered conditions. (a) Comparison of primary root lengths of 5- and 10-day-old DT2008 and W82 plants. (b) Comparison of numbers of secondary root of 5-day-old DT2008 and W82 plants. (c) Comparison of dry weights of root biomasses of 5-, 10-, 20-, and 25-day-old DT2008 and W82 plants. Error bars represent standard errors. For 5- and 10-d-old plants, *n* = 20 plants/genotype. For 20- and 25-d-old plants, *n* = 10 plants/genotype. Asterisks indicate significant differences as determined by a Student's *t*-test (*P < 0.05; ***P < 0.001).

To examine the correlation between the root development and enhanced drought-tolerant phenotype of DT2008 in comparison with W82, we first compared the root growth rate of DT2008 and W82 under well-watered conditions. For comparison under well-watered conditions, three root growth-related parameters, namely, the length of primary root, the number of secondary roots, and the DW of the whole root systems were evaluated. DT2008 plants were found to display better root development under normal conditions when compared with W82. The 5- and 10-day-old DT2008 plants have longer primary root and higher number of secondary roots than W82 (Figures 4(a) and 4(b)). Additionally, all the 5-, 10-, 20-, and 25-d-old DT2008 soybean plants possessed larger root total biomass than W82 plants as evidenced by their DW that was measured (Figure 4(c)).

**Figure 5:**
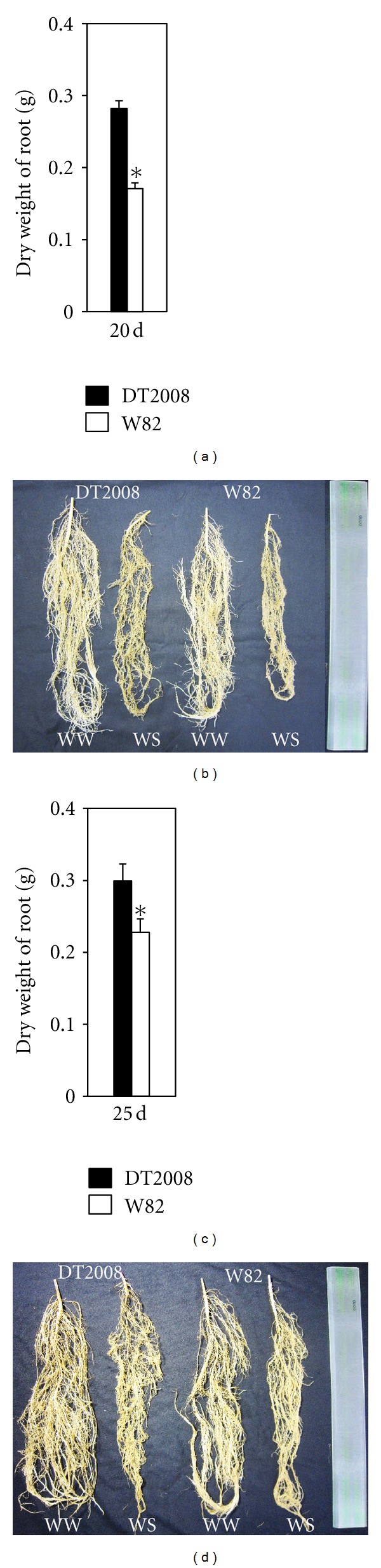
Comparison of root biomasses of DT2008 and W82 plants under drought stress. Five-day-old DT2008 and W82 plants grown in pots were exposed to 15 or 20 days of water withholding. (a) Comparison of dry weights of root biomasses of DT2008 and W82 plants after 15 days of drought stress. (b) Representative root samples of DT2008 and W82 plants grown under well-watered control or drought stress (15 days of stress) conditions. (c) Comparison of dry weights of root biomasses of DT2008 and W82 plants after 20 days of drought stress. (d) Representative root samples of DT2008 and W82 plants grown under well-watered control or drought stress (20 days of stress) conditions. Error bars represent standard errors (*n* = 10 plants/genotype). Asterisks indicate significant differences as determined by a Student's *t* test (*P < 0.05).

For the evaluation of root growth under drought stress conditions, the DWs of the total root biomasses of drought-stressed DT2008 and W82 plants were compared. DT2008 exhibited more sustainable root growth and development at reduced soil moisture conditions than W82 as much more notable reduction in the DW of roots was observed with W82 during drought stress treatment than with DT2008 (Figure 5). Collectively, our results suggest that the enhanced root systems of DT2008 may significantly contribute to its improved drought tolerance in relative to W82.

## 4. Conclusions

In this work, we have determined the differential drought tolerance phenotypes of DT2008 and W82 by comparing their capacity to maintain RWC as well as their shoot and root growths under normal, dehydration, and drought stress conditions. Our results indicated that DT2008 has stronger drought tolerance phenotype in comparison with W82. These two varieties can be used as genetic resources with contrasting drought-responsive phenotypes for the identification of mutations or mutated gene(s), which caused enhanced drought tolerance, using various omic approaches, such as transcriptomics, proteomics, and metabolomics, contributing to better understanding of drought response at molecular levels in soybean as well as providing candidate gene(s) for genetic engineering to improve drought tolerance of elite soybean cultivars. 

In addition, it will also be interesting to examine whether enhanced development of the root system of DT2008 contributes to the improved drought tolerance of DT2008, when compared with other previously used elite cultivars [6], in the field conditions as a major trait. Extensive work is currently undergoing to elucidate this relationship.
